# Proxy Information Seeking by Users of a Parenting Information Website: Quantitative Observational Study

**DOI:** 10.2196/32406

**Published:** 2022-03-04

**Authors:** Reem El Sherif, Pierre Pluye, Tibor Schuster, Roland Grad

**Affiliations:** 1 Department of Family Medicine McGill University Montreal, QC Canada

**Keywords:** consumer health information, information seeking behavior, child development, child health, information outcomes, health information, digital health, parenting, online information

## Abstract

**Background:**

One of the largest groups of consumers who seek health information on the internet are parents of young children, as well as people in their social circle. The concept of proxy seeking (on behalf of others) has been explored in the literature, yet little is known about the outcomes.

**Objective:**

The main aim of this study was to describe consumer health information outcomes reported by proxy seekers using a parenting website.

**Methods:**

We conducted a 2-year quantitative observational study. Participants were parents of 0- to 8-year-old children and members of their entourage in Canada who had accessed *Naître et Grandir* through the website or through a weekly newsletter. For each *Naître et Grandir* webpage, participants’ perceptions regarding the outcomes of seeking and using specific webpages were gathered using a content-validated Information Assessment Method questionnaire. We compared the outcomes reported by parents with those reported by members of their entourage after consulting a parenting information website and explored if the method of accessing the information by the proxy seekers (website or weekly newsletter) changed the outcomes reported. For key primary survey items, the chi-square test was conducted, and differences in relative frequencies of responses were computed along with confidence intervals.

**Results:**

A total of 51,325 completed questionnaires were included in the analysis, pertaining to 1079 *Naître et Grandir* webpages (mean 48; range 1-637). Compared to parents, individuals in the entourage are more likely to report using the information in discussion with others (mean difference 0.166, 95% CI 0.155-0.176). Parents, on the other hand, were more likely than the entourage to report using the information to better understand (mean difference 0.084, 95% CI 0.073-0.094), to decide to do something (mean difference 0.156, 95% CI 0.146-0.166), or to do something in a different manner (mean difference 0.052, 95% CI 0.042-0.061). In addition, results suggest that the differences in perceived benefits of parenting information by the entourage depend on how they access the information. Respondents who were actively seeking the information (through the website) were more likely to report that the information would help them be less worried (mean difference 0.047; 95% CI 0.024-0.069), handle a problem (mean difference 0.083; 95% CI 0.062-0.104), and decide what to do with someone else (mean difference 0.040, 95% CI 0.020-0.058). Respondents who passively acquired the information (through the newsletter) were more likely to report that the information would help improve the health or well-being of a child (mean difference 0.090; 95% CI 0.067-0.112).

**Conclusions:**

By better understanding how consumers and their entourages use information, information providers can adapt information to meet both individual and group needs, and health care practitioners can target patients’ entourages with web-based health information resources for dissemination and use.

## Introduction

### Background

In 2017, almost all (99.0%) Canadian households had fixed broadband internet access [1], and 75% to 96% of Canadians aged 15 to 64 years used the internet on a daily basis [2]. This is in line with other Organization for Economic Cooperation and Development (OECD) countries, in which more than 80% of households have access to high-speed internet [3]. In these countries, the proportion of adults seeking consumer health information on the internet has more than doubled between 2008 and 2017 [4]. The internet is a frequently accessed platform for finding consumer health information, in addition to common health information sources such as health care professionals or members of one’s social circle, and other sources such as books and television [5,6]. The use of trustworthy consumer health information from the internet can improve quality of life and is generally associated with increased empowerment of consumers and their families and with improved health outcomes [6-8].

There are, however, still barriers to benefitting from consumer health information from the internet. These include illness challenges, such as someone being too physically or mentally incapacitated to start a search for themselves. A second barrier may be lower eHealth literacy, meaning a consumer’s ability to seek, find, understand, and appraise consumer health information from the internet and apply the knowledge gained to addressing health issues. At least one-third of the population of 18 OECD countries may have low health literacy [9]. Moreover, when faced with a stressful situation, consumers may experience transitory low literacy level, as the interdependence between information and emotion is well established in the literature [10]. Finally, there are negative outcomes (or tensions) reported by users seeking consumer health information from the internet and health care practitioners.

Our recent qualitative study [11] described personal tensions, such as increased anxiety and interpersonal tensions between patients and physicians as a result of discussing consumer health information from the internet, and service-related tensions, such as postponing a clinical visit [11]. One strategy to reduce these tensions is discussing the information with someone in one’s social circle [11]. Approximately 90% of individuals in OECD countries report having access to social support (eg, relatives or friends) who can help them in times of need [12]. Access to social support is positively linked to internet access and use because these providers of support are internet users themselves and have relevant support and awareness [13]. Proxy consumer health information seeking on the internet is a common phenomenon: almost two-thirds of consumer health information seekers have reported searching on behalf of someone else to provide informational social support [14-16]. This proxy consumer health information seeking on the internet may overcome previously mentioned barriers. This is especially true if the support provider has higher eHealth literacy than the receiver: they are thus better able to explain, contextualize, or validate the information [17,18]. However, while there are several studies [19-21] that explored behavior related to proxy consumer health information seeking on the internet, few explored how the seeker uses the information with others, and what outcomes they report as a result of this use.

### Parents and Proxy Health Information–Seeking Behavior and Outcomes

One of the largest groups of consumers of web health information consumers is parents of young children. A recent systematic review [22] and empirical studies [23,24] on how parents find, use, and evaluate consumer health information from the internet for their children reported that parents worldwide are heavy users across diverse circumstances. Parents find the information themselves or reach out to their social circle (or entourage) for tailored advice, emotional support, and culturally relevant parenting information [25]. A 2015 survey, conducted in Quebec, of a representative sample of 23,693 parents of preschool children showed that only 1.5% of parents do not know where to find information on the internet about children, either directly or mediated by someone else [26] as a proxy—“seeking information in a nonprofessional or informal capacity on behalf (or because) of others without necessarily being asked to do so [27].”

Previous work [28] shows that the use of high-quality parenting consumer health information from the internet by mothers can lead to benefits such as decreased worries and increased self-confidence in decision-making, regardless of socioeconomic status [28]. However, little is known about proxy information seeking by the entourage of parents. The main objective of this study was to explore these outcomes as reported by users of a parenting information website. A secondary objective was to explore if the method of obtaining the information influences the reported outcomes of proxy information seeking on the internet.

## Methods

### Design

We conducted a 2-year quantitative observational study. Ethical approval was obtained from the institutional review board of the Faculty of Medicine, McGill University, prior to the start of data collection. We used the STROBE (Strengthening the Reporting of Observational Studies in Epidemiology) checklist [29].

### Consumer Health Information From the Internet Outcomes

**Figure 1:**
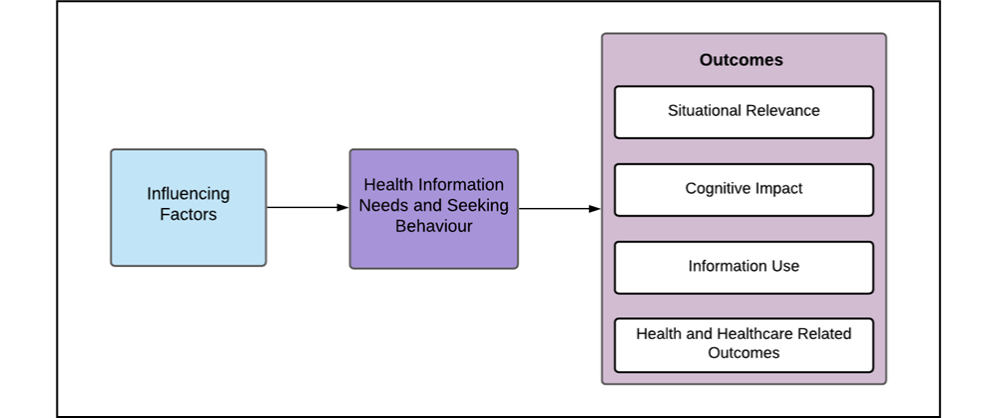
Outcomes framework.

A conceptual framework (Figure 1) was adapted from [30]. There are 4 types of influencing factors: individual characteristics (eg, age and income), sociotechnical factors (eg, eHealth literacy and social support), patient–professional relationships, and education–health–social resources. Together, these factors determine the extent to which information is accessed and how it is used by patients. An information need is a condition in which “certain information contributes to the achievement of a genuine or legitimate information purpose [31].” These needs may be explicitly stated or implicitly understood based on an individual’s health status or situation [31]. Seeking consumer health information on the internet is purposive and active searching for information as a consequence of an information need or to satisfy a goal [32]. Finally, there are 4 individual levels of web-based consumer health information–seeking outcomes: situational relevance, cognitive impact, and use of information, and health and health care–related outcomes.

### Naître et Grandir

The *Naître et Grandir* website provides parents with support in raising their children, from the time they are conceived until they are 8 years old.

*Naître et Grandir* is funded by the Lucie and André Chagnon Foundation, a philanthropic organization that seeks to contribute to the prevention of poverty through the creation of conditions and environments that are favorable to the educational success of children, specifically, those from socially vulnerable families and communities. Low health literacy levels in parents are detrimental to child health education, healthy behaviors, health, and medication, thus *Naître et Grandir* is an important resource for French-speaking parents and their entourage [33]. In addition to accessing the website directly, *Naître et Grandir* readers can sign up to receive a weekly newsletter containing links to *Naître et Grandir* webpages tailored to their child’s age. *Naître et Grandir* provides free parenting information content produced using an expert-based process and an editorial process that caters to lower health literacy levels (Grade 8 reading levels) with additional audio and video formats [28].

Since 2014, our team at McGill University and *Naître et Grandir* have worked in partnership to implement the Information Assessment Method questionnaire for evaluating the pages of parenting information. In addition, *Naître et Grandir* has been able to improve informational content based on the comments provided by Information Assessment Method users, which are coded by 2 editors in a web-based system. This is referred to as 2-way knowledge translation [34].

### Information Assessment Method

The framework is operationalized in the Information Assessment Method questionnaire used to evaluate health information outcomes from the viewpoint of information users (clinicians, managers, patients and public) [35]. The Information Assessment Method questionnaire has been content validated for different audiences using participatory mixed methods (integrating quantitative survey data with qualitative insights [36]). It has been implemented by different information providers to allow information users to rate specific health information content on the internet (eg, a webpage), stimulate their reflection, and collect feedback [35]. Consequently, responses and comments can be used by information providers to improve content.

**Figure 2:**
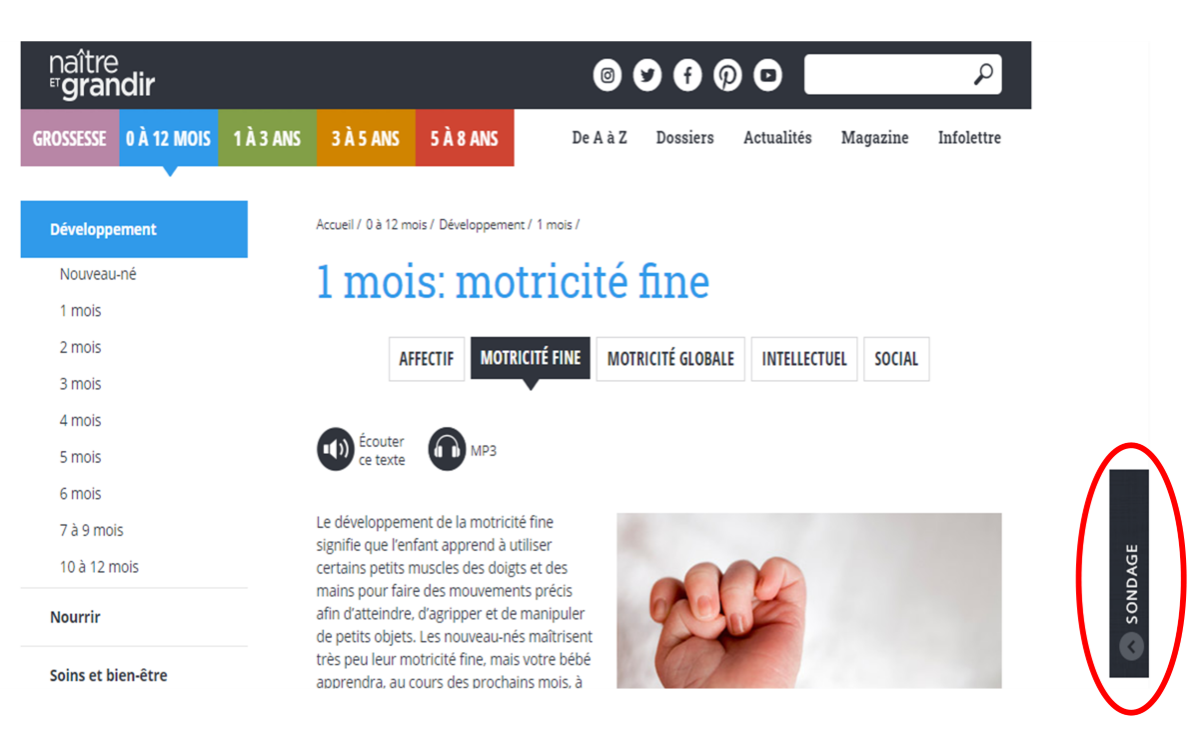
Screenshot from a *Naître et Grandir* page.

The validity of the Information Assessment Method has been assessed on 2 occasions: It was first validated specifically for parents in 2015 using quantitative data (raters’ responses) and qualitative data (raters’ comments and qualitative interviews [37]. It was then validated again in 2019 specifically for parents of lower socioeconomic status using qualitative data from interviews with low-socioeconomic status parents used in this study [28] and was validated in French (as it is implemented with *Naître et Grandir*) and underwent a transcultural adaptation into English (Multimedia Appendix 1). When *Naître et Grandir* readers land on a webpage corresponding to a specific topic (directly or through the newsletter link), a lateral tab appears inviting them to complete a survey (Figure 2).

### Study Participants and Data Collection

Data collection was co-constructed with *Naître et Grandir* in the course of the ongoing partnership. The editors of *Naître et Grandir* provided feedback on the questionnaire; however, they did not influence the data analysis and interpretation.

Participants in this study were *Naître et Grandir* readers in Canada and 4 other OECD countries with francophone populations (France, Belgium, Switzerland, and Luxembourg) that have similar health and social systems and comparable average household incomes, internet access, and reported social support levels [12].

Each participant had arrived at a specific *Naître et Grandir* webpage (either directly through the website), had clicked on the lateral tab, and had completed the Information Assessment Method questionnaire asking them to evaluate that specific *Naître et Grandir* webpage during the study period (April 13, 2019 to March 30, 2021). All completed questionnaires were included in the analysis. Among them, participants were divided into 2 group—self-identified parents of 0- to 8-year-old children or an entourage member (grandparent, family member, friend, neighbor, or professional working with children). A second analysis was conducted in the entourage group between those who had accessed the *Naître et Grandir* webpage and Information Assessment Method questionnaire through the weekly newsletter and those who had landed directly on the *Naître et Grandir* website. Variables included in the analysis correspond to the Information Assessment Method questions. No incentive was provided to participate.

### Statistical Analysis

Comparisons (1) between parents and entourage Information Assessment Method responses and (2) between newsletter and website Information Assessment Method responses from entourage were assessed using frequency analyses. Difference in proportions with 95% confidence intervals were calculated [38,39]. To take multiple comparisons into account and retain a global Type I error level of 5%, confidence levels were corrected using Bonferroni adjustment. In addition, the Pearson chi-square test was used to determine whether the differences between 2 groups of participants were statistically significant. Test results were deemed statistically significant when *P* values<.001. All statistical analyses were completed using SAS software (version 9.4; SAS Institute).

### Hypotheses

Based on our previous work exploring information outcomes, we hypothesized that, when the information is considered relevant and easy to understand, the entourage would be more likely to report discussing the information with others. We also hypothesized that, similar to previous work on parents’ responses, there would be a difference in entourage responses based on mode of access.

## Results

### All Respondents

**Figure 3:**
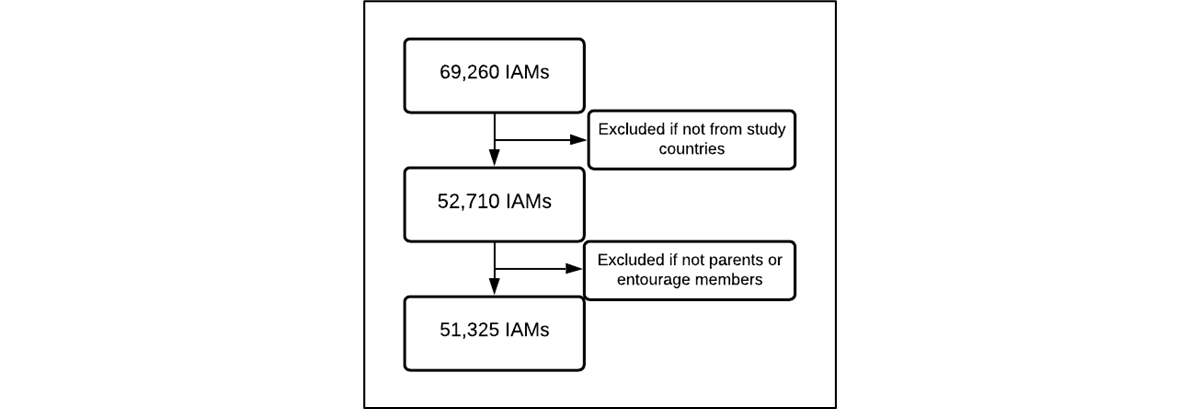
Information Assessment Method (IAM) questionnaires included in the analysis.

**Figure 4:**
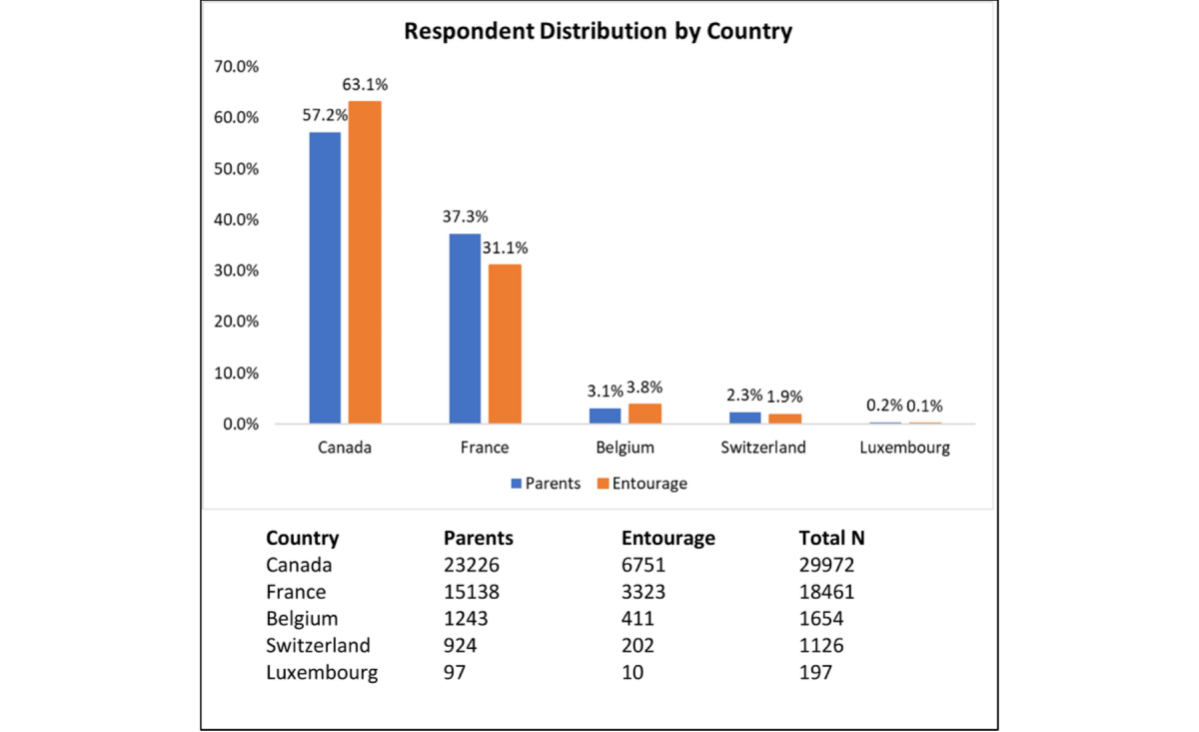
Respondent distribution by country.

**Figure 5:**
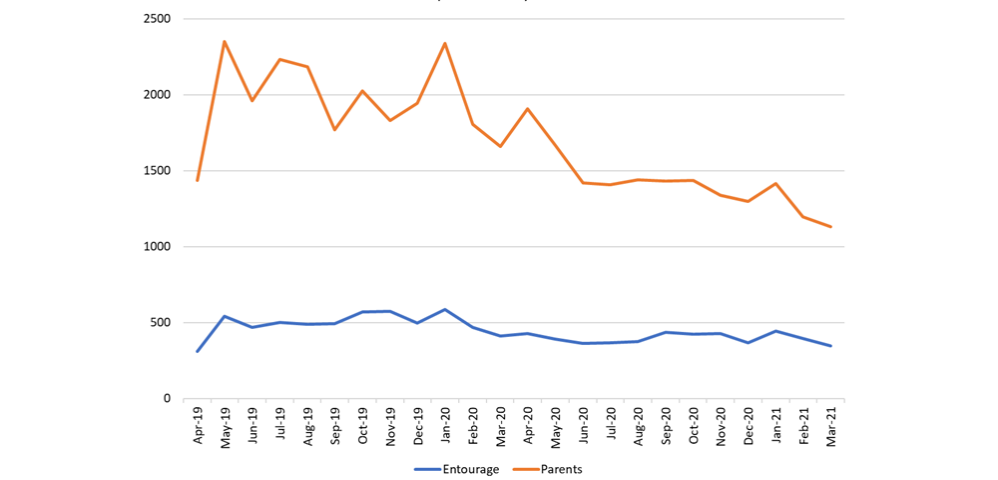
Response rate by month.

Over the 2-year study period, 69,260 Information Assessment Method questionnaires were completed. Questionnaires completed by participants outside the countries of interest in this study and by participants who did not identify as parents or entourage members were excluded (Figure 3). In total, 51,325 completed Information Assessment Method questionnaires were included in the analysis, pertaining to 1079 *Naître et Grandir* webpages (mean 48; range 1-637). Most respondents were in Canada (29,972/51,325, 58.4%) and France (18,461/51,325, 36%) (Figure 4). Parents comprised 79.2% (40,628/51,325) of participants, and grandparents were the most common entourage members (6309/51,325, 12.3%), followed by professionals, family, and friends (4388/51,325, 8.5%). The response rates of parents and entourage exhibited similar patterns (Figure 5).

### Comparing Parents and Entourage

Of the 51,325 Information Assessment Method questionnaires, 40,628 (79.2%) were completed by parents and 10,697 (20.8%) were completed by entourage members.

Parents were more likely to report using parenting information to better understand (mean difference 0.084, 95% CI 0.073-0.094), to decide to do something (mean difference 0.156, 95% CI 0.146-0.166), or to do something in a different manner (mean difference 0.052, 95% CI 0.042-0.061). They were also more likely to report that it helped them improve the health or well-being of a child (mean difference 0.039, 95% CI 0.028-0.049) and to be less worried (mean difference 0.104, 95% CI 0.093-0.114). The entourage members were more likely to use the information in discussion with someone else (mean difference 0.166, 95% CI 0.155-0.176) and report that the information helped them decide what to do with someone else (Table 1).

**Table 1:** Perceived information outcomes: Information Assessment Method responses of entourage members and parents.

Questions and response options			Entourage members (n=10,697), n (%)		Parents (n=40,628), n (%)		All participants (n=51,325), n (%)
**Q1. Is this information relevant?**							
	Very relevant (this is the information I expected)	7444 (69.6)		27,817 (68.5)		35,261 (68.7)	
	Relevant	2993 (27.9)		11,707 (28.8)		14,700 (28.6)	
	Somewhat relevant	123 (1.1)		654 (1.6)		777 (1.5)	
	Very little relevant (this is not the information I expected)	137 (1.3)		450 (1.1)		587 (1.1)	
**Q2. Did you understand this information?**							
	Very well (I understood everything)	9870 (92.3)		37,834 (93.1)		47,704 (92.9)	
	Well	777 (7.3)		2698 (6.6)		3475 (6.8)	
	Poorly	26 (0.2)		51 (0.1)		77 (0.2)	
	Very poorly (I did not understand much)	24 (0.2)		45 (0.1)		69 (0.1)	
**Q3. What do you think about this information?^a^**							
	This information allowed me to validate what I do or did	5611 (52.5)*		25,922 (63.8)		31,533 (61.4)	
	This information taught me something new	4753 (44.4)*		22,869 (56.3)		27,622 (53.8)	
	This information reassured me	2966 (27.7)*		17,037 (41.9)		20,003 (39.0)	
	This information refreshed my memory	3811 (35.6)*		9348 (23.0)		13,159 (25.6)	
	This information motivated me to learn more	2550 (23.8)*		8846 (21.8)		11,396 (22.2)	
	I do not like with this information	204 (1.9)		900 (2.2)		1104 (2.2)	
**Q4. Will you use this information?**							
	Yes	10,082 (94.3)		38,970 (95.9)		49,052 (95.6)	
	No	615 (5.8)		1658 (4.1)		2273 (4.4)	
**Q4a. How will you use this information for you or for a child in your care?^a^**							
	This information will help me to better understand.	4691 (46.5)		21,208 (54.4)		25,899 (52.8)	
	I will use this information to do something.	3637 (36.1)		20,143 (51.7)		23,780 (48.5)	
	I will use this information to do something in a different manner.	3026 (30.0)		13,585 (34.9)		16,611 (33.9)	
	I will use this information in a discussion with someone else.	4264 (42.3)		9473 (24.3)		13,737 (28.0)	
	I will use this information in another way.	356 (3.5)		760 (1.9)		1116 (2.3)	
**Q5. Using this information, do you expect any benefits for you and at least one child (0-8 years)?**							
	Yes	10,044 (93.9)		38,477 (94.7)		48,521 (94.5)	
	No	653 (6.1)		2151 (5.3)		2804 (5.5)	
**Q5a. Which benefits do you expect for you and at least one child (0-8 years)?^a^**							
	This information will help me to improve the health or well-being of my child.	6690 (62.5)		26,976 (66.4)		33,666 (65.6)	
	This information will help me to be less worried.	3480 (32.5)		17,424 (42.9)		20,904 (40.7)	
	This information will help me to prevent a problem or the worsening of a problem.	3184 (29.8)		12,406 (30.5)		15,590 (30.4)	
	This information will help me to handle a problem.	3226 (30.2)		12,966 (31.9)		16,192 (31.6)	
	This information will help me decide what to do with someone else.	2137 (20.0)		5597 (13.8)		7734 (15.1)	
	Another benefit.	408 (3.8)		871 (2.1)		1279 (2.5)	

^a^More than 1 option could be selected.

### Comparing Website and Newsletter Respondents

Of 10,697 Information Assessment Method questionnaires completed by the entourage, 1953 (18.3%) accessed the webpage through the newsletter and 8744 (81.7%) directly through the website. Respondents through the newsletter were more likely to report using the information to do something (mean difference 0.117, 95% CI 0.092-0.141) or do something differently (mean difference 0.067, 95% CI 0.044-0.090) and expected that the information would help to improve the health or well-being of a child (mean difference 0.090; 95% CI 0.067-0.112). Respondents who accessed *Naître et Grandir* directly through the website were more likely to report that using the information would help them be less worried (mean difference 0.047; 95% CI 0.024-0.069), handle a problem (mean difference 0.083; 95% CI 0.062-0.104), and decide what to do with someone else (mean difference 0.040, 95% CI 0.020-0.058). Both groups were equally likely to report using the information in discussion with someone else (mean difference 0.015; 95% CI –0.009-0.040) (Table 2).

**Table 2:** Perceived information outcomes: Information Assessment Method responses of entourage newsletter and website respondents.

Questions and response options			Entourage newsletter (n=1953), n (%)		Entourage website (n=8744), n (%)		All entourage (n=10,697), n (%)
**Q1. Is this information relevant?**							
	Very relevant (this is the information I expected)	1547 (79.2)		5897 (67.4)		7444 (69.6)	
	Relevant	390 (20.0)		2603 (29.8)		2993 (28.0)	
	Somewhat relevant	7 (0.4)		116 (1.3)		123 (1.2)	
	Very little relevant (this is not the information I expected)	9 (0.5)		128 (1.5)		137 (1.3)	
**Q2. Did you understand this information?**							
	Very well (I understood everything)	1891 (96.8)		7979 (91.3)		9870 (92.3)	
	Well	59 (3.0)		718 (8.2)		777 (7.3)	
	Poorly	1 (0.1)		25 (0.3)		26 (0.2)	
	Very poorly (I did not understand much)	2 (0.1)		22 (0.3)		23 (0.2)	
**Q3. What do you think about this information?^a^**							
	This information allowed me to validate what I do or did	1118 (57.3)		4493 (51.4)		5611 (52.5)	
	This information taught me something new	898 (46.0)		3855 (44.1)		4753 (44.4)	
	This information reassured me	519 (26.6)		2447 (28.0)		2966 (27.7)	
	This information refreshed my memory	839 (43.0)		2972 (34)		3811 (35.6)	
	This information motivated me to learn more	427 (21.9)		2123 (24.3)		2550 (23.8)	
	I do not like with this information	29 (1.5)		175 (2.0)		204 (1.9)	
**Q4. Will you use this information?**							
	Yes	1902 (97.4)		8180 (93.6)		10,082 (94.3)	
	No	51 (2.6)		564 (6.5)		615 (5.8)	
**Q4a. How will you use this information for you or for a child in your care?^a^**							
	This information will help me to better understand.	865 (45.5)		3826 (46.8)		4691 (46.5)	
	I will use this information to do something.	850 (44.7)		2787 (34.1)		3637 (36.1)	
	I will use this information to do something in a different manner.	659 (34.7)		2367 (28.9)		3026 (30.0)	
	I will use this information in a discussion with someone else.	754 (39.6)		3510 (42.9)		4264 (42.3)	
	I will use this information in another way.	53 (2.8)		303 (3.7)		356 (3.5)	
**Q5. Using this information, do you expect any benefits for you and at least one child (0-8 years)?**							
	Yes	1891 (96.8)		8153 (93.2)		10,044 (93.9)	
	No	62 (3.2)		591 (6.8)		653 (6.1)	
**Q5a. Which benefits do you expect for you and at least one child (0-8 years)?^a^**							
	This information will help me to improve the health or well-being of my child.	1365 (69.9)		5325 (60.9)		6690 (62.5)	
	This information will help me to be less worried.	561 (28.7)		2919 (33.4)		3480 (32.5)	
	This information will help me to prevent a problem or the worsening of a problem.	605 (31.0)		2579 (29.5)		3184 (29.8)	
	This information will help me to handle a problem.	456 (23.4)		2770 (31.7)		3226 (30.2)	
	This information will help me decide what to do with someone else.	327 (16.7)		1810 (20.7)		2137 (20.0)	
	Another benefit.	77 (3.9)		331 (3.8)		408 (3.8)	

^a^More than 1 option could be selected.

## Discussion

### Principal Findings

Results support our first hypothesis that individuals in the entourage were more likely to report using the information in discussion with others. Parents, on the other hand, were more likely to report using the information to do something. This may reflect the trustworthiness of the information on *Naître et Grandir*—the entourage felt comfortable sharing it and parents feel comfortable applying it.

Our second hypothesis was also supported. Results suggest that the differences in perceived outcomes reported by the entourage depend on how they access the information. When the information is acquired through active seeking by the respondents through the *Naître et Grandir* website, there were differences in the reported use and benefits. These findings can be explained by the literature on information seeking behavior (Table 3), specifically Bates’s integrated model of information seeking [40], in which, there are 2 forms of information seeking: directed, through searching and monitoring when there is a known information need, and undirected, through browsing and being unaware when the information need is unknown.

**Table 3:** Applying of the integrated model of information seeking [40] to this study's context.

Form	*Naître et Grandir* website	*Naître et Grandir* newsletter
Information need	Known	Unknown
Information seeking mode	Directed and active	Undirected and passive

In our study, respondents arrived on the website through directed and active searching that was likely triggered by a known information need such as an existing health problem. The entourage members responding through the website were also aware of the information need by the parents, either because it was stated explicitly by the parents or understood implicitly through social interactions. The entourage members in this context may have closer social ties and may be involved in the decision making, either directly or indirectly, by providing social support. The entourage in this group were thus more likely to report that the information they found would help them to be less worried, help them handle a problem, and help them decide what to do with someone else. On the other hand, entourage respondents through the newsletter were less likely to have a known information need and would have clicked on one of the relevant articles out of interest or curiosity (undirected and passive information seeking). This group was more likely to report that the information would help them improve the health or well-being of a child.

### Comparison With Prior Work

We identified the role of known and unknown information needs on the outcomes of proxy information seeking by entourage members, by comparing entourage website and newsletter users. This is the first unique contribution of our study, as most similar studies [14-16,19] have focus on directed consumer health information seeking on the internet triggered by a known information need. Our study also describes these outcomes from the entourage or proxy seeker’s perspective. Another study [11], which explored negative outcomes of seeking consumer health information on the internet from the individual’s perspective, reported that in situations wherein informational support from the entourage is unsolicited and the individual does not feel that the information is relevant to their situation, interpersonal tensions between both parties may develop.

We explored the phenomenon of proxy consumer health information seeking using an evidence-based web-based consumer health information source that caters to lower health literacy. Thus, common barriers to positive outcomes such as health literacy and misinformation were somewhat removed, and we could describe the outcomes experienced by parents and their entourage in this context. A recent scoping review [41], which explored parents’ web-based health information–seeking behaviors to inform vaccination choices for their children, reported significant misinformation on the topic on the internet and suggested parents’ digital health literacy may influence their decisions.

Our results are transferable to other contexts. While we do not claim statistical generalizability as the study sample was self-selected, respondents were not limited by demographic criteria and thus represent a diverse sample of parents and their entourage. Moreover, our respondents rated webpages presenting a wide number of health and well-being topics (ie, not focused on any specific illness or condition). A recent systematic review [22], which explored health information seeking on the internet by parents for their children, identified lack of generalizability as the most frequently mentioned limitation of the studies included in the review. In fact, an agenda item for future research studies was the need for studies with generalizable samples outside clinical environments with specific populations of children who are ill [22]. While the review [22] explored parent health information seeking on the internet as a form of proxy seeking, their findings do not apply to other types of proxy seeking [22]. In this study, we provided insight into another type of proxy seeking and the reported outcomes.

### Limitations

Our study has 3 main limitations. First, participants were self-selected volunteers who completed one questionnaire at one point of time (a source of selection bias). This likely led to an overestimation of positive outcomes due to social desirability bias [42]. However, this bias will have influenced both parents and the entourage in the same manner and thus did not affect the comparative analysis. Moreover, we cannot assume website users and newsletter users were mutually exclusive. Second, we did not explore variables such as the strength of the social ties between the entourage and the parents and child for whom they were using *Naître et Grandir*. Other studies [16,43,44] have reported that proxy information seekers are likely to have strong ties with the people they are helping and tend to provide other forms of social support such as emotional support. This limitation will be addressed in a future study with entourage members.

### Conclusions

The results will be used to refine and improve the existing conceptual framework on consumer health information outcomes on the internet by filling in the gap on the role of the information need. When information is acquired through active directed seeking by the respondents from the *Naître et Grandir* website, they were likely to use it and report positive outcomes related to an existing problem. When information is acquired from the weekly newsletter, respondents were more likely to report more general positive outcomes. Regardless of how they accessed information, members of the entourage were likely to discuss it with others. Practical intervention strategies can focus on improving proxy health information seeking on the internet and extend social support networks for people without an effective entourage. Future studies can explore how members of the entourage use the information from *Naître et Grandir* with others in their social circle.

## Appendix

Multimedia Appendix 1 Information Assessment Method questionnaire.

## Abbreviations

OECD: Organization for Economic Cooperation and Development
