# Supplementary material for: Proxy Information Seeking by Users of a Parenting Information Website: Quantitative Observational Study
Source: JMIR Pediatr Parent. 2022 Mar 4;5(1):e32406. doi: 10.2196/32406 (PMC8933803; doi:10.2196/32406)
Supplement: Multimedia Appendix 1 [file pediatrics_v5i1e32406_app1.docx]

**Appendix. IAM4parents-v2019 questionnaire**

**You are responding mainly as a:**

- Pregnant woman or mother of a child (0-8 years)
- Partner of a pregnant woman or mother of a child (0 to 8 years old)
- Grandparent of a child (0-8 years)
- Other family member of a child (0-8 years)
- Friend, neighbor or relative of a child (0-8 years)
- Professional caring for children (0-8 years)
- Other response (Comment)

**Q1. Is this information relevant? (Choose only one)**

- Very relevant (this is the information I expected)
- Relevant
- Somewhat relevant
- Very little relevant (this is not the information I expected)

**Q2. Did you understand this information? (Choose only one)**

- Very well (I understood everything)
- Well
- Poorly
- Very poorly (I did not understand much)

**Q3. What do you think about this information? (Check all that apply)**

- This information allowed me to validate what I do or did
- This information taught me something new
- This information reassured me
- This information refreshed my memory
- This information motivated me to learn more
- I do not like with this information

**Q4. Will you use this information? (Choose only one)**

- Yes
- No

**Q4a. How will you use this information for you or for a child in your care? (Check all that apply)**This information will help me to better understand.

- I will use this information to do something.
- I will use this information to do something in a different manner.
- I will use this information in a discussion with someone else.
- I will use this information in another way.

**Q5. Using this information, do you expect any benefits for you and at least one child (0-8 years)? (Choose only one)**

- Yes
- No

**Q5a. Which benefits do you expect for you and at least one child (0-8 years)? (Check all that apply)**

- This information will help me to improve the health or well-being of my child.
- This information will help me to be less worried.
- This information will help me to prevent a problem or the worsening of a problem.
- This information will help me to handle a problem.
- This information will help me decide what to do with someone else.
- Another benefit.
